# CausNet-partial : ‘Partial Generational Orderings’ based search for optimal sparse Bayesian networks via dynamic programming with parent set constraints

**DOI:** 10.21203/rs.3.rs-4021074/v1

**Published:** 2024-03-07

**Authors:** Nand Sharma, Joshua Millstein

**Affiliations:** Division of Biostatistics, Department of Population and Public Health Sciences, University of Southern California, Los Angeles, USA.

**Keywords:** Optimal Bayesian Network, dynamic programming, generational orderings

## Abstract

**Background:**

In our recent work, we developed a novel dynamic programming algorithm to find optimal Bayesian networks (BNs) with parent set constraints. This ‘generational orderings’ based dynamic programming search algorithm - CausNet - efficiently searches the space of possible BNs given the possible parent sets. The algorithm supports both continuous and categorical data, as well as continuous, binary and survival outcomes. In the present work, we develop a variant of CausNet - CausNet-partial - which searches the space of ‘partial generational orderings’, which further reduces the search space and is suited for finding smaller sparse optimal Bayesian networks; and can be applied to 1000s of variables.

**Results:**

We test this method both on synthetic and real data. Our algorithm performs better than three state-of-art algorithms that are currently used extensively to find optimal BNs. We apply it to simulated continuous data and also to a benchmark discrete Bayesian network ALARM, a Bayesian network designed to provide an alarm message system for patient monitoring. We first apply the original CausNet and then CausNet-partial varying the partial order from 5 to 2. CausNet-partial discovers small sparse networks with drastically reduced runtime as expected from theory.

**Conclusions:**

Our partial generational orderings based search for small optimal networks, is both an efficient and highly scalable approach for finding optimal sparse and small Bayesian Networks and can be applied to 1000s of variables. Using specifiable parameters - correlation, FDR cutoffs, in-degree, and partial order - one can increase or decrease the number of nodes and density of the networks. Availability of two scoring option - BIC and Bge - and implementation for survival outcomes and mixed data types makes our algorithm very suitable for many types of high dimensional data in a variety of fields.

## 1 Introduction

Optimal Bayesian Network (BN) Structure Discovery is a method of learning Bayesian networks from data that has applications in wide variety of areas including epidemiology (see e.g. [1], [2], [3], [4], [5], [6]). Disease pathways found using BN leading to a phenotype outcome can lead to better understanding, diagnosis and treatment of a disease. The challenge in finding an optimal BN lies in the super-exponential complexity of the search. Dynamic programming can reduce the complexity to exponential, but still the number of features/nodes being explored remains very small—on the order of 10 or 20 nodes—and only by restricting the maximum number of parents for each node. To alleviate the challenge of high dimensionality in GWAS and other such datasets, we developed CausNet - a dynamic programming approach on the space of ‘Generational orderings’ with parent set restrictions in [7]. This ‘generational orderings’ based search for optimal networks is a novel way to efficiently search the space of possible networks given the possible parent sets.

Current algorithms typically do not accommodate both continuous and categorical nodes, and we were not able to find any that accommodate a survival outcome. We implemented support for both continuous and categorical data, as well as continuous, binary and survival outcomes. This is especially useful for disease modeling where mixed data and survival outcomes are common. We also provide options for two common scoring functions, and allow for multiple best networks to be returned if there are ties.

Our main novel contribution in addition to providing software is the revision of the Silander algorithm 3 [8] to incorporate possible parent sets, and use of ‘generational orderings’ for a much more efficient way to explore the search space as compared to the original approach, which is based on lexicographical ordering. The proposed approach covers the entire constrained search space without searching through networks that don’t conform to the parent set constraints.

In the present work, we extend Causnet to further optimize the search for smaller and sparse BNs using what we call ‘Partial Generational orderings’. Here we are interested in finding only a small number of variables and a small causal network leading to an outcome of interest. This is a common scenario in many domains with high-dimensional data where we are interested in a much smaller set of variables as biomarkers having a causal effect on the disease outcome. The subspace of ‘Partial Generational orderings’ can reduce the search space drastically and can be applied to large-dimensional data. The amount of reduction in search space can be controlled by the user according to the problem and prior domain knowledge.

## 2 Background

A Bayesian network (BN) ([9], [10]) is a probabilistic graphical model that consists of a labeled directed acyclic graph (DAG) in which the vertices $V=\left\{v_{1},\ldots ,v_{p}\right\}$ correspond to random variables and the edges represent conditional dependence of one random variable on another. Each vertex $v_{i}$ is labeled with a conditional probability distribution $P\left(v_{i}|\right.\;parents\left.\left(v_{i}\right)\right)$ that specifies the dependence of the variable $v_{i}$ on its set of parents $parents\left(v_{i}\right)$ in the DAG.

A Bayesian network G can be represented by a vector $G=\left(G_{1},\ldots ,G_{p}\right)$ of parent sets: $G_{i}$ is the subset of V from which there are directed edges to $v_{i}$, i.e. elements of $G_{i}$ are the parents of the node $v_{i}$ in G. Any G that is a DAG corresponds to an ordering of the nodes, say the ordered set $\left\{v_{\sigma_{i}}\right\},\;i\in \{1,\;2,..,p\}$, where σ is a permutation of [p] - the ordered set of first p natural numbers, with $\sigma (i)=\sigma_{i}$. A BN is said to consistent with an ordering $\left\{v_{\sigma_{i}}\right\}$ if parents of $v_{\sigma_{i}}$ are a subset of $\left\{v_{\sigma_{j}}\right\},\;j<i$, i.e. $G_{\sigma_{i}}\subseteq \left\{v_{\sigma_{j}}\right\},\;j<i$.

One of the main methods for BN structure learning from data uses a scoring function that assigns a real value to the quality of G given the data. For finding a best network structure, we maximize this score over the space of possible networks. Note that we can have multiple best networks with the same score. Scoring functions balance goodness of fit to the data with a penalty term for model complexity. Some commonly used scoring functions are BIC/MDL [11], [12], [13], BDeu [14], [15], and BGe [15], [16], [17]. We use BIC (Bayesian information criterion) and BGe (Bayesian Gaussian equivalent) scoring functions as two options for using Causnet and CausNet-partial. These two scoring functions have been shown to perform consistently well for large data, and for discrete and continuous variables ([13], [18]). BIC is a log-likelihood (LL) score where the overfitting is avoided by using a penalty term for the number of parameters in the model, specifically pln(n), where n is the sample size. The BGe score is the posterior probability of the model hypothesis that the true distribution of the set of variables is faithful to the DAG model, meaning that it satisfies all the conditional independencies encoded by the DAG, and is proportional to the marginal likelihood and the graphical prior[15] [16] [17].

## 3 CausNet

CausNet [7] uses the dynamic programming (DP) approach to finding a best Bayesian network structure for a given dataset following the work by Koivisto & Sood [19], [20], Silander and Myllymäki in [8] and by Singh and Andrew in [21].

Finding a best Bayesian network structure is NP-hard [22], [23]. The number of possible structures for n variables is $𝒪\left(n!2\left(\begin{array}{c} n\\ 2 \end{array}\right)\right)$ [24], making exhaustive search impractical. So the dynamic programming algorithms are feasible only for small data sets; usually less than 30 variables ([8], [21]); CausNet closely follows the algorithm proposed by Silander and Myllymäki [8] (SM algorithm henceforth) and makes heuristic modifications to reduce the search space and scales it up for higher number of variables. This is achieved by parent set identification, and by restricting the search to the space of ‘generational’ orderings rather than lexicographical ordering as in the original SM algorithm. Furthermore, CausNet supports continuous, binary as well as survival data and provides two options of scores: BIC and BGe.

The dynamical programming approach uses the key fact about DAGs that every DAG has at least one sink. The problem of finding a best Bayesian network given the data 𝒟 starts with finding a best sink for the whole set of nodes. Then that node is removed and the process is repeated recursively for the remaining set of nodes. The result is an ordering of the nodes $\left\{v_{\sigma_{i}}\right\},\;i\in \{1,\;2,..,p\}$, from which the DAG can be recovered. Denoting the best sink by s, and the score of a best network with nodes V by bestscore(V), and the best score of s with parents in U by bestScore(s,U), the recursion is given by the following relation :

(1) bestScore(V)=bestscore(V\{s})+bestScore(s,V\{s}).

To implement the above recursion, the idea of a local score for a node $v_{i}$ with parents $parents\left(v_{i}\right)$ is used, which we get using a scoring function. The requirement for a score function score(G) for a network G is that it should be decomposable, meaning that the total score of the network is the sum of scores for each node in the network, and the score of a node depends only on the node and its parents. Formally,

(2) $$score\left(G\right)=\sum_{i=1}^{p}localscore\left(v_{i},G_{i}\right).$$

where the local scoring function localscore(x,y) gives the score of x with parents y in the network G. In a given set of possible parents $pp_{i}$ for node $v_{i}$, we find the best set of parents $bps_{i}$ which give the best local score for $v_{i}$, so that

(3) $$\left.bestScore\left(v_{i},pp_{i}\right)\right)=\underset{g\subseteq pp_{i}}{max}\;localscore\left(v_{i},g\right),$$

(4) $${bps}_{i}\left(pp_{i}\right)=\underset{g\subseteq pp_{i}}{argmax\;}localscore\left(v_{i},g\right).$$

Now the best sink s can be found by Eq. 5, and the best score for a best network in V can be found by Eq. 6.


(5) $$bestSink(V)=\underset{s\in V}{argmax}\;bestscore(V\;\backslash \;\{s\})+bestScore(s,V\;\backslash \;\{s\}).$$

(6) $$bestscore(V)=\underset{s\in V}{max}\;\;bestscore(V\;\backslash \;\{s\})+bestScore(s,V\;\backslash \;\{s\}).$$

**Figure 1:**
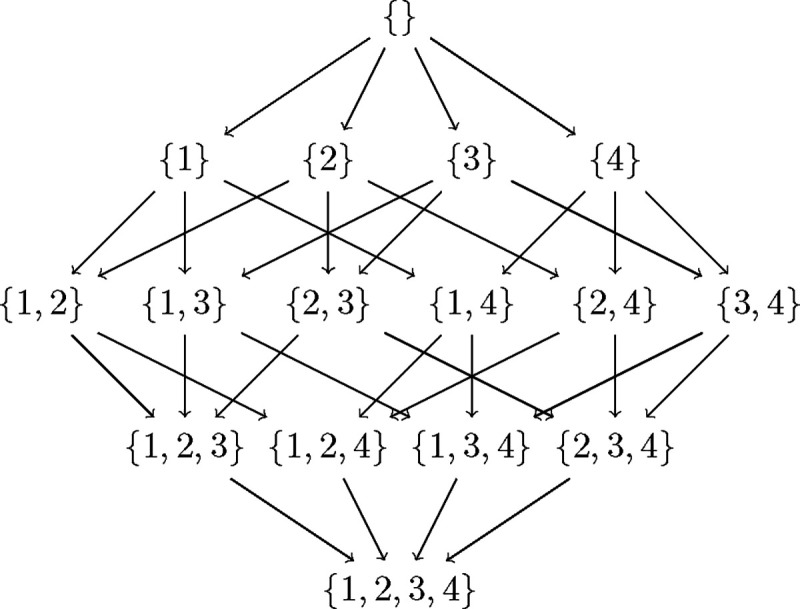
Subset lattice on a network with four nodes {1, 2, 3, 4}.

**Figure 2:**
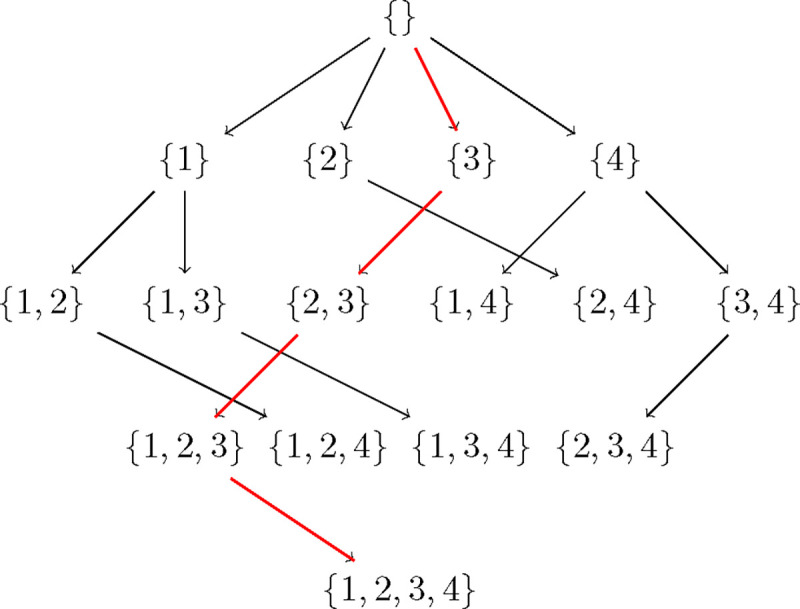
An example Best sinks sublattice of four nodes {1, 2, 3, 4}. The arrows encode the best sink for each subset. The red arrows indicate the best network given by the only fully connected path, and corresponds to the reverse-ordering {4, 1, 2, 3}.

In Figure 1 as an example, the subset lattice of four nodes {1, 2, 3, 4} shows all the paths that need to be searched to find a best network. Observe that each edge in the lattice encodes a sink - if $p_{s}$ is the subset at the source and $p_{h}$ the subset at the end of a directed edge, the corresponding sink is given by $p_{h}\backslash p_{s}$. Each path in the subset lattice encodes an ordering on the 4 variables, e.g. the rightmost path encodes the reverse-ordering {1, 2, 3, 4}. There are a total of 4! paths/orderings to be searched. Now suppose we knew the best score corresponding to each edge in all the paths, meaning the best score for the sink corresponding to that edge with the best parents from the subset at the source of that edge. Then naive depth/width first search would compare the sum of scores along all paths to get the best network. In dynamic programming, we proceed from the top of the lattice. Ignoring the empty set, we start with finding the best sink for each singleton in the first row which trivially is the singleton itself. Next, we find the best sink for the subsets of cardinality 2 in the second row using the edge best scores. And we continue all the way down. Suppose we get the best sinks sublattice as in Figure 2, then the best network is given by the only fully connected path, and corresponds to the reverse-ordering {4, 1, 2, 3}.

Now, using the SM algorithm, finding the best Bayesian network structure, also the basic CausNet approach without restricting the search space, has the following five steps:

1. Calculate the local scores for all $p2^{p-1}$ different (variable, variable set)-pairs.
2. Using the local scores, find best parents for all $p2^{p-1}$ (variable, possible parent set)-pairs.
3. Find the best sink for all $2^{p}$ variable sets.
4. Using the results from Step 3, find a best ordering of the variables.
5. Find a best network using results computed in Steps 2 and 4.

The extensions to this base version of CausNet - possible parent sets identification, phenotype driven search, and search space based on ‘Generational orderings’ reduce the search space. Here we reproduce the algorithms of the CausNet method. For details, the reader is referred to [7].

**Figure 3:**
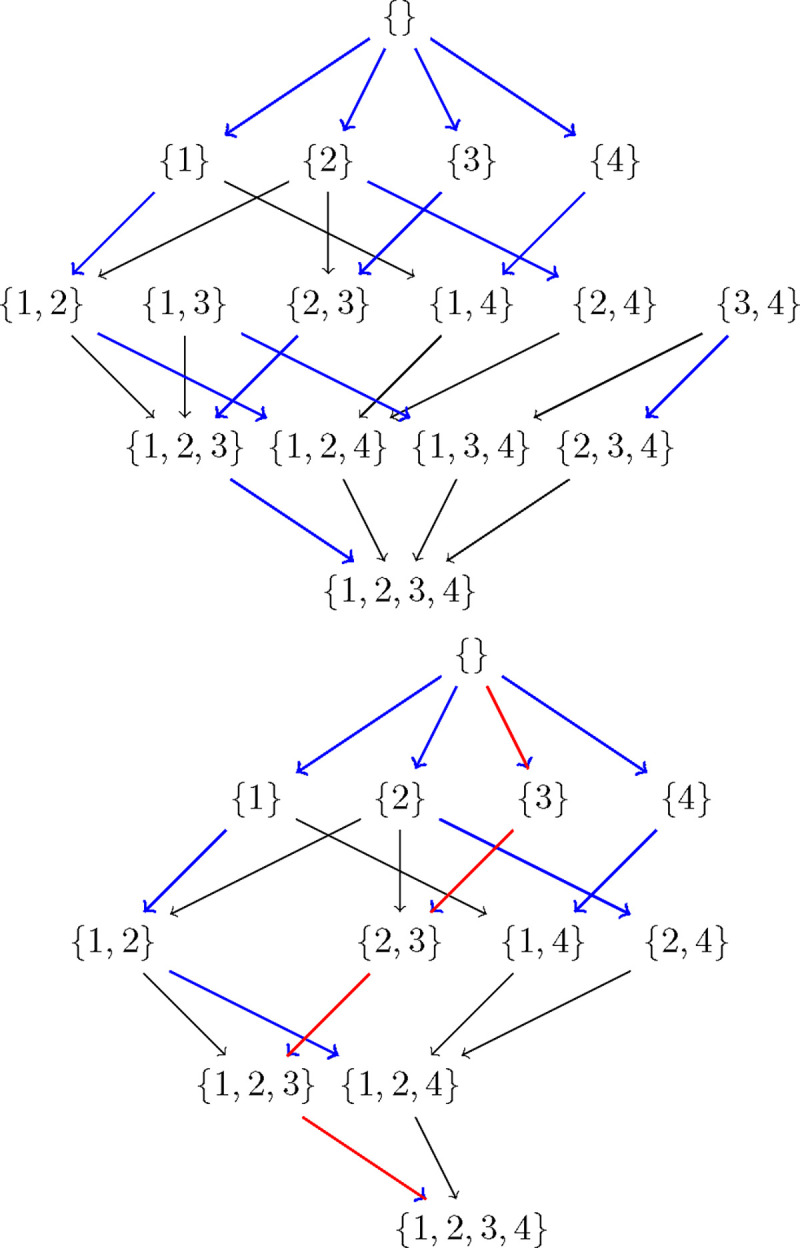
Subset lattices with parent set constraints. The top subset lattice is the lattice with parent set restrictions. The bottom lattice, obtained by Causnet, retains only the subsets that are in the complete generational orderings. The red arrows which at base are blue as well, represent the best network.

As an example of how CausNet works, suppose the possible parent sets for the four nodes {1, 2, 3, 4} are as follows : $pp_{1}=\left\{2,\;4\right\},pp_{2}=\{3,\;1\}$, $pp_{3}=\{2\}$, $pp_{4}=\{1\}$. Factoring in these possible parent sets, the subset lattices corresponding to those in figures 1 and 2 reduce to those in Figure 3. Here we are showing the lattices with parent set constraints, so that some arrows are omitted e.g. in the top lattice, there is no arrow from {4} to {3, 4} because node 3 does not have node 4 as a possible parent in our example. The bottom lattice retains only the subsets that are in the complete path from the top of the lattice to its bottom, and discards the remaining paths. These full paths represent what we call *complete generational orderings*. This is how generational ordering of Causnet ensures maximum connectivity among the reduced set of $\overset{ˆ}{p}$ nodes in the *feasSet*. The red arrows which at base are blue as well, represent the best network.

**Algorithm 1:** Find pp, Compute feasSet and feasSetData

	**Input** : Data, *α*, phenotypeBased, pp
1:	**if** pp Not NULL **then**
2:	pp = pp
3:	**else**
4:	**if** phenotypeBased =True **then**
5:	find pp for the phenotype output
6:	find pp for the phenotype’s pp
7:	**else**
8:	find pp for all variables
9:	**end if**
10:	**end if**
11:	find possible offsprings (po) for all variables
12:	find the feasible set of variables *feasSet*
13:	get the reduced dimensional data *feasSetData*
	**Output** : pp, po, feasSet and feasSetData

**Algorithm 2:** Compute local scores for feasSet nodes

	**Input** : feasSetData, pp
1:	**for** *v_i_* in *feasSet* **do**
2:	find all parent subsets {*pps_j_*} of possible parents set *pp_i_* of the node *v_i_*
3:	compute local score for node *v_i_* with parents *pps_j_*
4:	**end for**
	**Output** : pps, ppss

**Algorithm 3:** Compute best scores and best parents for feasSet nodes in all parent subsets

	**Input** : feasSetData, pps, ppss, indegree
1:	**for** *v_i_* in *feasSet* **do**
2:	**for** *p_ij_* in *pps_i_* **do**
3:	find all subsets {*ppsSub_ijk_*} upto cardinality indegree
4:	Compute best scores *bpss_i_j* and best parents *bps_ij_* of *v_i_* for parent subset *pps_ij_* from among {*ppsSub_ijk_*} using local scores in *ppss*
5:	**end for**
6:	**end for**
	**Output** : pps, ppss, bps, bpss

**Algorithm 4:** Compute best sinks for feasSet subsets

	**Input** : feasSetData, po, pps, ppss, bps
1:	Start with $\overset{‾}{p}$ networks of single nodes, where $\overset{‾}{p}$ is the number of nodes in *feasSet*, with their NULL scores
2:	Add one offspring at a time, and iterate over all nodes in the set to find a best sink using information about best score/parent set from algorithm 3; end when number of nodes in the iteration is $\overset{‾}{p}$
3:	**return** list of all possible $2^{\overset{‾}{p}}$ subsets of *feasSet* with best sink and best network score for that subset of nodes
	**Output** : bsinks

**Algorithm 5:** Find the best networks

	**Input** : bsinks
1:	Find the reverse ordering (possibly multiple orderings) of $\overset{‾}{p}$ nodes using the list from algorithm 4
2:	Compute best network(s) using the reverse ordering(s) of $\overset{‾}{p}$ nodes and using the best parent set for each node in a possible parent set found in algorithm 3
	**Output** : bestNetwork(s)

## 4 CausNet-partial - Dynamic programming on the space of ‘partial generational orderings’

In Causnet-partial, we introduce the space of ‘partial generational orderings’ of nodes instead of full generational orderings to find sparse optimal sparse BNs. In this approach we start not at the level of singletons in the algorithm for finding best sinks as is done in basic CausNet but at a certain level of subsets of cardinality $\overset{‾}{p}-pOrd$, where pOrd is the length of partial orderings that we want to consider. For example, if we want to search partial orderings of length 2 for our example of 4 variables, we will start at the level of subsets of cardinality 2 instead of one. This reduces the search space of orderings, and is useful when we are interested in sparse networks.

**Figure 4:**
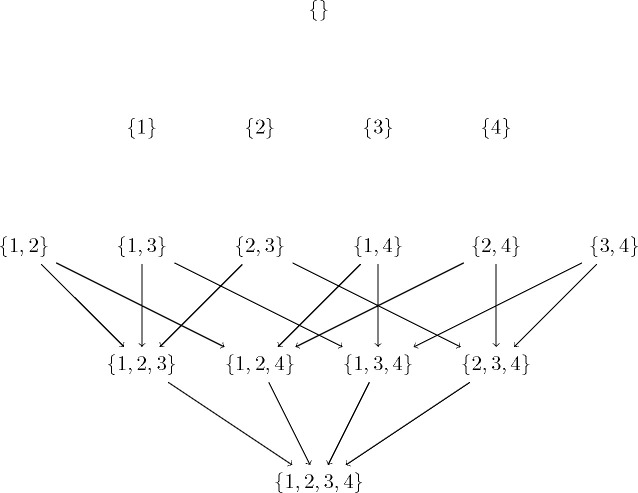
Subset lattice for partial orderings with pOrd=2 on four nodes {1, 2, 3, 4}.

The bestSinks algorithm of basic CausNet (algorithm 4) is modified to algorithm 6 in case of CausNet-partial. The subset lattice to be explored for partial orderings of length 2 for our example of 4 variables becomes as in figure 4. Search for sparse networks in the space of Partial Orderings reduces the run time of the best sinks algorithm substantially, and thus of the CausNet-partial method.

**Algorithm 6:** Compute best sinks for feasSet subsets - partial orderings

	**Input** : feasSetData, po, pps, ppss, bps
1:	Start with subsets of cardinality $\overset{‾}{p}-pOrd$
2:	Find best sink for each subset of cardinality $\overset{‾}{p}-pOrd$
3:	Add one offspring at a time, and iterate over all nodes in the set to find a best sink using information about best score and best parent set; end when number of nodes in the iteration is $\overset{‾}{p}$
4:	**return** list of all possible subsets of *feasSet* with cardinality $\overset{‾}{p}-pOrd$ up to cardinality $\overset{‾}{p}$ with best sink and best network score for that subset of nodes
	**Output** : bsinks

### Theorem 4.1

Total number of labeled DAGs with p nodes and partial order r is $𝒪\left(\frac{p!}{(p-r)!}\frac{2^{\left(\begin{array}{c} p\\ 2 \end{array}\right)}}{2^{\left(\begin{array}{c} p-r-1\\ 2 \end{array}\right)}}\right)$.

#### Proof

For p nodes, total number of orderings is p!; With pOrd=r, the number of orderings with r nodes is $\frac{p!}{(p-r)!}$. For each node in the partial ordering of r nodes, the number of possible parent sets is given by-

$$\begin{array}{r} 2^{\left(p-1\right)}2^{\left(p-2\right)}\cdots 2^{\left(p-r-1\right)}\\ \;=\frac{2^{\left(\begin{array}{c} p\\ 2 \end{array}\right)}}{2^{\left(\begin{array}{c} p-r-1\\ 2 \end{array}\right)}}. \end{array}$$

So, the Total number of labeled DAGs with p nodes and partial order r is given by :

$$\frac{p!}{(p\;-\;r)!}\frac{2^{\left(\begin{array}{c} p\\ 2 \end{array}\right)}}{2^{\left(\begin{array}{c} p-r-1\\ 2 \end{array}\right)}}.$$

The number of network structures is $𝒪\left(\frac{p!}{(p-r)!}\frac{2^{\left(\begin{array}{c} p\\ 2 \end{array}\right)}}{2^{\left(\begin{array}{c} p-r-1\\ 2 \end{array}\right)}}\right)$ because there are many repeated structures in this combinatorial computation; e.g. there are (r+1)! structures with all (r+1) nodes disconnected.

### Theorem 4.2

Without parent set and in-degree restrictions, the CausNetpartial -the partial generational ordering based DP algorithm - explores all the $𝒪\left(\frac{p!}{(p-r)!}\frac{2^{\left(\begin{array}{c} p\\ 2 \end{array}\right)}}{2^{\left(\begin{array}{c} p-r-1\\ 2 \end{array}\right)}}\right)$ network structures for p nodes.

#### Proof

Without parent set restrictions, every node is s possible parent of every other node. In Figure 1, let k,0≤k≤p be the cardinality of subsets in the subset lattice for p nodes. Let each row in the subset lattice be the *k*th level in the lattice. Now adding a new element in algorithm 4 corresponds to an edge between a subset of cardinality k and k−1, which considers the added element as a sink in the subset of cardinality k. Number of edges to a subset of cardinality k from subsets of cardinality k−1 is given by $\left(\begin{array}{c} k\\ k-1 \end{array}\right)$. The number of possible parent combinations for a sink in subset of cardinality k, without in-degree restrictions, is given by $2^{k-1}$. In algorithm 3, we explore all these possible parent sets to find the best parents for each sink s in each subset at each level k. The algorithm 6 uses this information to get the best sink (possibly multiple) for each subset at level k. Thus the total number of networks thus searched by the Causnet-partial algorithm is given by -

$$\begin{array}{l} \;\prod_{k=(p-r)}^{p}\;\;\left(\begin{array}{c} k\\ k-1 \end{array}\right)2^{k-1}\\ \;=\left(\begin{array}{c} p-r-1\\ p-r \end{array}\right)\left(\begin{array}{c} p-r+2\\ p-r+1 \end{array}\right)\ldots \left(\begin{array}{c} p-1\\ p-2 \end{array}\right)\left(\begin{array}{c} p\\ p-1 \end{array}\right)2^{p-r-1}2^{p-r}\ldots 2^{p-1}\\ \;=\frac{p!}{(p\;-\;r)!}\frac{2^{\left(\begin{array}{c} p\\ 2 \end{array}\right)}}{2^{\left(\begin{array}{c} p-r-1\\ 2 \end{array}\right)}}\text{.} \end{array}$$

The number of network structures is $𝒪\left(\frac{p!}{(p-r)!}\frac{2\left(\begin{array}{c} p\\ 2 \end{array}\right)}{2^{\left(\begin{array}{c} p-r-1\\ 2 \end{array}\right)}}\right)$ because there are many repeated structures in this combinatorial computation; e.g. there are (r+1)! structures with all (r+1) nodes disconnected.

## 5 Simulations

We compare CausNet and CausNet-partial with three other methods that have been widely used for optimal Bayesian network identification to infer disease pathways from multiscale genomics data. The first method is Bartlett and Cussens’ GOBNILP [25], an integer learning based method that’s considered state-of-art exact method for finding optimal Bayesian network. The other two methods are BNlearn’s Hill Climbing (HC) and Max-min Hill Climbing (MMHC) ([26], [27]), which are both widely used approximate methods, see e.g. [28], [29]. Hill-Climbing (HC) is a score-based algorithm that uses greedy search on the space of the directed graphs [30]. Max-Min Hill-Climbing (MMHC) is a hybrid algorithm [31] that first learns the undirected skeleton of a graph using a constraint-based algorithm called Max-Min Parents and Children (MMPC); this is followed by the application of a score-based search to orient the edges.

We simulated Bayesian networks by generating an N×p data matrix of continuous Gaussian data. The dependencies are simulated using linear regression with the option to control effect sizes. Some number of the p nodes were designated as sources $\left(p_{1}\right)$, some intermediate $\left(p_{2}\right)$, and some sinks $\left(p_{3}\right)$, the remainder $\left(p_{0}\right)$ being completely independent. The actual DAGs of the $p_{1}+p_{2}+p_{3}$ nodes vary across replicates. The requirement for being a DAG is implemented using the idea of ordering of vertices. We pick a random ordering of a randomly chosen subset of p vertices. Then enforcing each vertex to have parents only from the set of vertices above itself in the ordering guarantees a DAG.

The False Discovery Rate (FDR) and Hamming Distance are used as the metrics to compare the methods. With FP defined as the number of false positives and TP defined as the number of true positives, FDR is defined as :

$$FDR=\frac{FP}{FP\;+\;TP}.$$

Controlling for the false discovery rate (FDR) is a way to identify as many significant features (edges in case of BNs) as possible while incurring a relatively low proportion of false positives. This is especially useful metric for high dimensional data and for network analysis ([32]).

The Hamming distance between two labeled graphs $G_{1}$ and $G_{2}$ is given by $|\left\{\left(\left(e\in E\left(G_{1}\right)\&\left(e\notin E\left(G_{2}\right)\right)\right)\right.\right.\;or\;\left.\left(\left(e\notin E\left(G_{1}\right)\&e\in E\left(G_{2}\right)\right)\right)\right\}|$, where $E\left(G_{i}\right)$ is the edge set of graph $G_{i}$. Simply put, this is the number of addition/deletion operations required to turn the edge set of $G_{1}$ into that of $G_{2}$. The Hamming distance is a measure of structural similarity, and forms a metric on the space of graphs (simple or directed), and gives a good measure of goodness of a predicted graph ([33], [34]). In the context of predicted and the truth graph, with FP defined as the number of false positives and FN as the number of false negatives, Hamming Distance is defined as :

HammingDistance=FP+FN.

As the first set of simulations using parent set identification, we ran simulations using multiple replicates of networks, the first with p=10,20,40,50,60,100, and N=500, 1000, 2000. For using CausNet and CausNet-partial, we have used FDR cutoff of 0.3 and an in-degree of 2 for all the experiments. In [7], we have shown how CausNet compares with the three other methods. Here we include CausNetpartial in the two tables 1 and 2. We use pOrd=3 for search on partial orderings in CausNet-partial. In the tables 1 and 2, we show the average FDR across the 9 combinations of N and p, both with and without taking directionality into account. The results for CausNet and CausNet-partial with the BIC scoring function are given. The results show that our method performs very well compared with the methods considered.

For the number of variables up to 40 (Table 1), on the metric of FDR, CausNet-partial performs the best, while Gobnilp is the second best and Causnet the third best for directed graphs. For undirected graphs, CausNet-partial and CausNet are the first and second best performers followed by Gobnilp. For the number of variables between 50 and 100 (Table 2), our methods are the top two best for both directed and undirected graphs.

**Figure 5:**
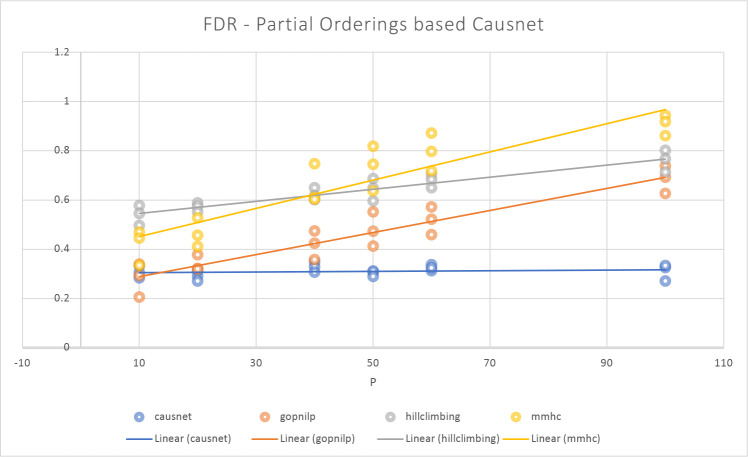
Average FDR with p=10,20,40,50,60,80,100 and N=500,1000,2000. Here the CausNet uses Partial orderings based search and uses parent set identification for dimensionality reduction while other algorithms don’t use any parent set identification.

**Figure 6:**
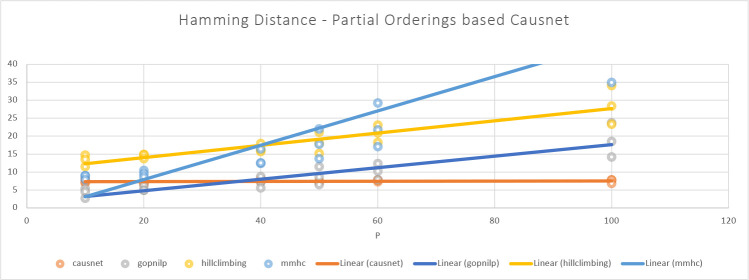
Average Hamming Distance with p=10,20,40,50,60,80,100 and N=500,1000,2000. Here the CausNet uses Partial orderings based search and uses parent set identification for dimensionality reduction while other algorithms don’t use any parent set identification.

The figures 5 and 6 show the comparison of CausNet-partial with the three methods on the metrics of FDR and Hamming distance. The FDR for Partial Orderings based Causnet (CausNet-partial) is the best for number of variables greater than 10; Gobnilp is the best for 10 variables and the second best for number of variables greater than 10. In terms of Hamming distance, CausNet-partial again is the best for variables more than 20; Gobnilp and mmhc have lower Hamming distance than that of Causnet for variables less than 20, but rise quickly for variables more than 40; overall, Gobnilp is again the second best. An interesting thing is that the linear trend for both FDR and Hamming distance for CausNet-partial is horizontal. This is because the simulated networks had three levels of nodes starting from the outcome node, and this is identified with high accuracy by Partial Orderings based Causnet with pOrd=3.

### 5.1 Number of variables upto 1000

**Figure 7:**
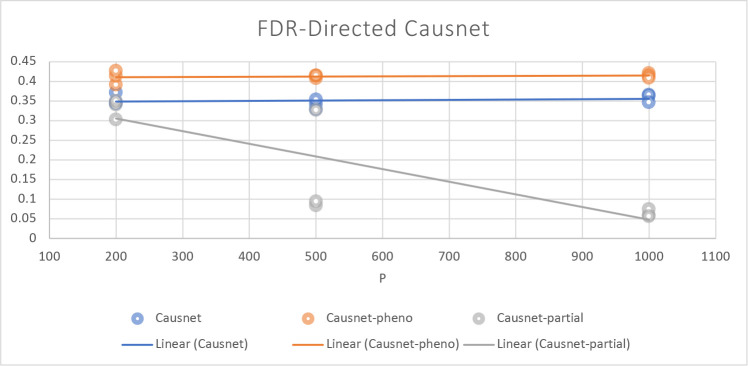
Average FDR with p=200,500,1000 and N=500,1000,2000. Here we compare the three versions of Causnet - Causnet basic, Phenotype based Causnet and Partial Orderings Causnet.

**Figure 8:**
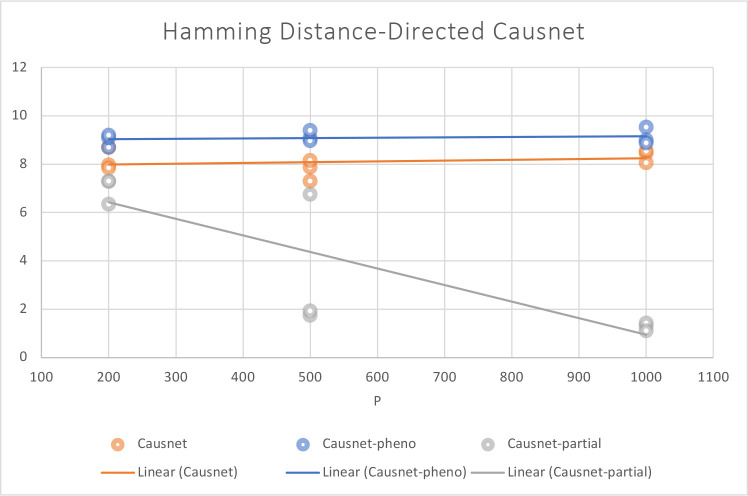
Average Hamming Distance with p=200,500,1000 and N=500,1000,2000. Here we compare the three versions of Causnet - Causnet basic, Phenotype based Causnet and Partial Orderings Causnet.

For these simulations, we use p=200,500,1000, and N=500,1000,2000. For these simulations, we don’t compare with the other three algorithms as they either can not handle such high number of variables or take many orders of magnitude longer. Instead, we compare the three variants of CausNet - CausNet [7], phenotypedriven CausNet [7], and CausNet-partial. The results are shown in figures 7 and 8. Here we observe that Partial Orderings Causnet gives the best results for both FDR and Hamming distance. Furthermore, both the FDR and Hamming Distance for Partial Orderings Causnet keeps getting better as the number of variables increase. This can be explained by the fact that as we reduce the length of orderings being searched there is a lesser chance of false positives. This also shows the effectiveness of using partial orderings where we expect sparse networks while the original data has much higher number of features, a common scenario for high dimensional data.

### 5.2 Runtime

Search for sparse networks in the space of Partial Orderings reduces the run time of the best sinks algorithm substantially, and thus of the Causnet-partial method. Having confirmed the performance of CausNet and Causnet-partial as superior to other algorithms, especially for the number of variables greater than 40, we compare the runtimes of the five algorithms. Here we split the comparison into two categories - number of variables less than 100 and greater than 100, i.e *p* ≤ 100 and 100 < *p* ≤ 1000. This is done as two of the algorithms - MMHC and Gobnilp - either can not handle more than 100 variables or the computation time is many orders of magnitude greater than that taken by CausNet and Causnet-partial. Specifically, MMHC takes over an average of 300 minutes, and Gobnilp terminates without producing any output network in most cases. Running all the simulations for these two algorithms would require an inordinate amount of time and was not considered worth the effort. The average runtimes in seconds are summarized in Table 3. As we can clearly see, Causnet-partial and Causnet have the top best runtimes for *p* ≤ 100 and Gobnilp the worst. For 100 < *p* ≤ 1000, Causnet-partial and CausNet are better than Gobnilp and MMHC. Although HC is faster, sections above demonstrated it to be inferior to CausNet in terms of performance metrics of FDR and Hamming distance. Between Causnet-partial and CausNet, Causnet-partial gives substantially reduced runtime, further validating the efficacy of Causnet-partial for large dimensional data.

## 6 Application to benchmark Bayesian network - ALARM

**Figure 9:**
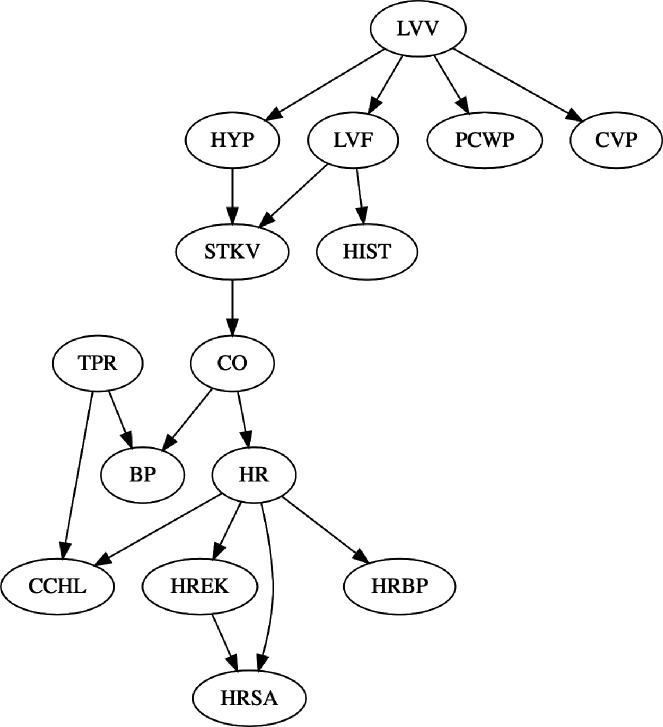
BN discovered by CausNet

**Figure 10:**
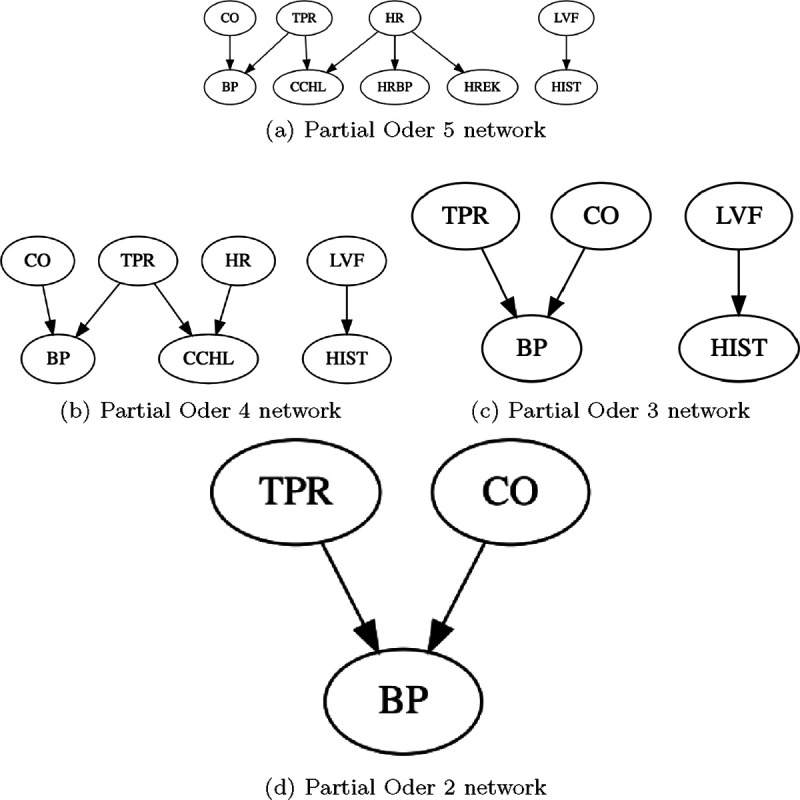
ALARM networks with different partial orderings

Having shown the better performance of CausNet and CausNet-partial, we show the efficacy of our method in finding sparse BNs now based on partial orderings. To this end, we apply our method to a benchmark Bayesian network ALARM [35]. The ALARM (“A Logical Alarm Reduction Mechanism”) is a Bayesian network designed to provide an alarm message system for patient monitoring. The data set contains 37 variables, all discrete, with number of categories between 2 and 4. We first apply the original CausNet with discrete option for BIC score. Then we apply CausNet-partial varying the partial order from 5 to 2. Fig. 9 shows the BN discovered by CausNet, and Fig. 10 shows the BNs discovered by CausNet-partial with different partial orders. CausNet discovers a BN with 15 nodes when using the in-degree 2 and the FDR cut-off of 0.3 for possible parent set identification. With the same in-degree and the FDR cut-off, we then apply the CausNet-partial to obtain sparser BNs based on partial generational orderings. The results are shown in Table 4. As we can see from the figures and the table, we get smaller and smaller networks as we reduces pOrd. The FDR for CausNet-partial is a bit higher than the one we get with CausNet, but this keeps getting better as pOrd is reduced. FDR for undirected networks is consistently 0 for CausNet-partial. Initial rise in FDR is due to substantial reduction of nodes from CausNet to CausNet-partial. The nodes and the directed edges in the partial order 5 network are subsets of nodes and the directed edges in the CausNet network. The lower partial order networks maintains the same consistency with the directed edges. The BIC score of the optimal networks increases as expected as the number of nodes and edges decrease in the networks. The gain in speed of computation is illustrated well with the runtime decreasing by many orders of magnitude going from CausNet to CausNet-partial.

## 7 Discussion

In this work, we implemented an extension to our earlier method CausNet. This extension - CausNet-sparse - is a dynamic programming based optimal Bayesian network (BN) structure discovery algorithm with parent set identification with ‘partial generational orderings’ based search for optimal networks. CausNet-sparse is a novel way to efficiently search the space of possible small and sparse networks given the possible parent sets.

Our main novel contribution aside from providing software is the revision of the SM algorithm 3 [8] to incorporate possible parent sets and ‘partial generational orderings’ based search for a more efficient and cost-effective way to explore the search space as compared to the original approach based on lexicographical ordering. This enables our method to be applied to large dimensional data effectively, while the SM algorithm can not be applied to more than 30 variables [8]. In doing so, we cover the entire constrained search space without searching through networks that don’t conform to the parent set constraints and the partial order. While the basic algorithm CausNet can be applied to any dataset from any domain, the CausNet-sparse is particularly suitable for finding small networks leading to an outcome of interest.

The simulation results show that our algorithm performs very well when compared with three state-of-art algorithms that are widely used currently. The parent set constraints and partial orderings reduce both the search space and the runtime significantly, while delivering better results, especially for greater than 20 variables. We further show its application it to a benchmark discrete Bayesian network ALARM, a Bayesian network designed to provide an alarm message system for patient monitoring. We first apply the original CausNet and then CausNet-partial varying the partial order from 5 to 2. CausNet-partial discovers small sparse networks with drastically reduced runtime as expected from theory developed as part of this work.

Important features of the algorithm include specifiable parameters - correlation, FDR cutoffs, in-degree, and partial order - which can be tuned according to the application domain. Choice of two scoring options, BIC and Bge, and implementation of survival outcomes and mixed data types makes CausNet-sparse suitable for identifying small disease pathways from a broad range of biomedical data types, e.g. GWAS and other omics.

## Figures and Tables

**Table 1:** FDR (*p* = 10, 20, 40, *N* = 500, 1000, 2000)

Method	FDR(undirected)	FDR(directed)

CausNet	0.229	0.412
CausNet-partial	**0.22**	**0.32**
Gobnilp	0.312	0.345
BN-HC	0.466	0.577
BN-MMHC	0.368	0.511

**Table 2:** FDR (*p* = 50, 60, 100, *N* = 500, 1000, 2000)

Method	FDR(undirected)	FDR(directed)

CausNet	0.359	0.494
CausNet-partial	**0.22**	**0.32**
Gobnilp	0.540	0.560
BN-HC	0.635	0.694
BN-MMHC	0.787	0.812

**Table 3:** Average Runtime in seconds.

	*p* ≤ 100	100 < *p* ≤ 1000

CausNet	0.025	277.87
CausNet-partial (pOrd 3)	0.018	123.7
Gobnilp	99.8	*
HC	0.088	68.17
MMHC	0.288	18763.493

* Terminates without output.

**Table 4:** FDR and Runtime for ALARM

	No. of nodes	FDR (directed)	FDR (undirected)	BIC score	Runtime (secs)

CausNet	15	0.35	0.05	−2913.6	153
CausNet-partial (pOrd 5)	9	0.55	0	−557.75	41.6
CausNet-partial (pOrd 4)	7	0.42	0	−484.84	21.9
CausNet-partial (pOrd 3)	5	0.2	0	−414.18	16.6
CausNet-partial (pOrd 2)	3	0	0	−136.85	15.9

## Data Availability

The CausNet software package with in-built CausNet-sparse in R is available at https://github.com/nand1155/CausNet.
